# Supplementary material for: Patterns of Intron Gain and Loss in Fungi
Source: PLoS Biol. 2004 Nov 30;2(12):e422. doi: 10.1371/journal.pbio.0020422 (PMC532390; doi:10.1371/journal.pbio.0020422)
Supplement: Table S1 — Also available at http://genes.mit.edu/NielsenEtAl/. (4.3 MB ZIP). [file pbio.0020422.st001.zip › NielsenEtAl/html/904.html]

AN7051.1.NCU07987.1.MG07074.1.FG01932.1


```
 CLUSTAL W (1.82) Multiple Sequence Alignments - Introns Inserted


Sequence 1: MG07074.1	454 aa
Sequence 2: FG01932.1	436 aa
Sequence 3: NCU07987.1	457 aa
Sequence 4: AN7051.1	458 aa
Alignment Length: 467 aa
Number Identitical Residues: 279 aa
Alignment Score (without introns) 12373


MG07074.1 	MAGQPS-IPSAI~GAKKALGLVDVDGHDLPPSPAPSSPSNGRRRYALATELVYTETKDQY
NCU07987.1	MSTPNSDSPAAQ~AAKKVFSRLDLDGHNLPPSPAPSSPHNGR-RYALATELVYTETKDQY
FG01932.1 	MASPSP-TESGN2-----------EGHDLPPSPAPSSPRTGRKRYALATELVYTDSKDQY
AN7051.1  	MSASSTLKKAFP~-------QVDAEGHNLPPSPAPSSPHGSR-RYNIATELVYTESNDQY
          	*:   .   :            . :**:**********  .* ** :*******:::***

MG07074.1 	GASSIPIYQ~SATFKQSSAAGGNSEYDYTRSGNPTRTHLERHMAKIMNANRALAVGSGMG
NCU07987.1	GASSIPIYQ~SATFKQSSSNGG-SEYDYTRSGNPTRTHLERHLAKIMNANRCLSVSSGMG
FG01932.1 	GASSVPIYQ~SATFKQTSASGGQQEYDYTRSGNPTRTHLERHIAKIMNAQRALAISSGMG
AN7051.1  	NASSVPIYQ0SATFKQSSHEGG-GEYDYTRSGNPTRTHLERHLAKIMSAQRALVVSSGMA
          	.***:**** ******:*  **  ******************:****.*:*.* :.***.

MG07074.1 	ALDVITRLLRPGDEVITGDDLYGGTNRLLTYLSANQGIIVHHIDTTDIDRVAATVSDKTA
NCU07987.1	ALDVITRLLKPGDEVITGDDLYGGTNRLLTYLKNNQGVIVHHVDTTNVESVRQIISPKTT
FG01932.1 	ALDVITRLLRPGDEVITGDDLYGGSHRLLTYLAANQGIVVHHVDTTTVDSVRARLSEKTA
AN7051.1  	ALDVITRLLRPGDEVVTGDDLYGGTNRLLKYLSTNGGIIVHHVDTTNPDKVKEVLTDKTA
          	*********:*****:********::***.**  * *::***:***  : *   :: **:

MG07074.1 	MVLLETPTNPLIKIVDVATIARAAHDRNSKALVVVDNTMLSPMLCNPLDLGADIVYESGT
NCU07987.1	MVLLETPTNPLIKICDIPTIARITHEANEKAVVVVDNTMLSPMLFNPLDVGADIVYESGT
FG01932.1 	MVLLETPTNPLIKVVDIPSIARLVHDVNPKALVVVDNTMLSPMLFNPLDVGCDIVYESGT
AN7051.1  	MVLLETPTNPLIKIVDIPTIAAASHEANPNCLVIVDNTMMSPLLLSPLELGADVVYESGT
          	*************: *:.:**   *: * :.:*:*****:**:* .**::*.*:******

MG07074.1 	KYLSGHHDIMSGIVACNDAALADRMYFTVNATGCGLSPNDSFLLMRGIKTLAVRMEKQQA
NCU07987.1	KYLSGHHDIMAGVIAVNDTELGDKLYFTINATGCGLSPNDSFLLMRGVKTLAIRMEKQQA
FG01932.1 	KYLSGHHDIMAGVIAVNDSQIGDKLFFVINSTGCGLSPNDSFLLMRGVKTLAIRMEKQQA
AN7051.1  	KYLSGHHDVMAGVIAVNDPALGERLYFPINASGCGLSPFDSWLLMRGVKTLKVRMDQQQA
          	********:*:*::* **. :.::::* :*::****** **:*****:*** :**::***

MG07074.1 	NAQRIAEFLESHGFRVRYPGLKSHPQYDLHWSMARGAGAVLSFETGDAELSQRIVEAARL
NCU07987.1	NAQRIAEFLESHGFKVRYPGLKSHPQYDLHWSMARGAGAVLSFETGDVALSERIVEAARL
FG01932.1 	NAQAIAEFLESRGFRVRYPGLKSHPQYDLHWSMARGAGAVLSFETGDPAVSERIVEAARL
AN7051.1  	NAQRIAEFLESHGFKVRYPGLRSHPQYDLHHSMARGAGAVLSFETGDVSVSERIVANAKL
          	*** *******:**:******:******** ****************  :*:***  *:*

MG07074.1 	WAISVSFGCVNSLISMPCQMSHASIDAKTRKERQMPEDIIRLCVGIEDVQDLIDDLSRA0
NCU07987.1	WGISVSFGCVNSLISMPCRMSHASIDAKTRAERQMPEDIIRLCVGIEDADDLIDDLSRA0
FG01932.1 	WAISVSFGCVNSLISMPCQMSHASIDAKTRAERQMPEDIIRLCVGIEDPTDLIEDLSRA0
AN7051.1  	WAISVSFGCVNSLISLPCRMSHASIDAKTRKERAMPEDLIRLCVGIEDVDDLIDDLRRA~
          	*.*************:**:*********** ** ****:*********  ***:** ** 

MG07074.1 	LVTAG-----AVSITVDGFHAT----GTALELGQTPLTTTKNDSPAQ---
NCU07987.1	LVQAG-----AVTLTVDGFHANTAEGAAAAAAGSEAAETTTTAAPSL---
FG01932.1 	LVQAG-----AVTVTMDGFHAT----GAAKELGETPLVFQ----------
AN7051.1  	VSVSTRNRVFWVMLIFSSWYKLALSTSLWTALKRTRRSLRSQVMKEYGYG
          	:  :  .    * : ...::  : . .             .      . .
```
